# Impact of a youth-friendly HIV clinic: 10 years of adolescent outcomes in Port-au-Prince, Haiti

**DOI:** 10.7448/IAS.19.1.20859

**Published:** 2016-07-04

**Authors:** Lindsey K Reif, Rachel Bertrand, Charles Benedict, Matthew R Lamb, Vanessa Rouzier, Rose Verdier, Warren D Johnson, Jean W Pape, Daniel W Fitzgerald, Louise Kuhn, Margaret L McNairy

**Affiliations:** 1 Center for Global Health, Weill Cornell Medical College, New York, NY, USA; 2 Department of Epidemiology, Mailman School of Public Health, Columbia University, New York, NY, USA; 3 Groupe Haitien d'Etude du Sarcome de Kaposi et des Infections Opportunistes (GHESKIO), Port-au-Prince, Haiti; 4 Gertrude H. Sergievsky Center, College of Physicians and Surgeons, Columbia University, New York, NY, USA; 5 Division of Hospital Medicine, Department of Medicine, Weill Cornell Medical College, New York, NY, USA

**Keywords:** adolescent, attrition, epidemiology, HIV/AIDS, retention, youth

## Abstract

**Introduction:**

Adolescents account for over 40% of new HIV infections in Haiti. This analysis compares outcomes among HIV-positive adolescents before and after implementation of an adolescent HIV clinic in Port-au-Prince, Haiti.

**Methods:**

We conducted a cohort study using programmatic data among HIV-positive adolescents aged 13 to 19. Data from 41,218 adolescents who were HIV tested from January 2003 to December 2012 were included. Outcomes across the HIV care cascade were assessed before and after implementation of an adolescent clinic (2009), including HIV testing, enrolment in care, assessment for a*ntiretroviral therapy* (ART) eligibility, ART initiation and 12-month retention. Pre-ART outcomes were assessed 12 months after HIV testing. Factors associated with pre-ART and ART attrition were identified through multivariable competing risk and Cox proportional hazards regression modelling.

**Results:**

Cumulatively, 1672 (4.1%) adolescents tested HIV positive (80% female, median age 16 years). Retention by cascade step comparing pre- and post-clinic included the following: 86% versus 87% of patients enrolled in care, 61% versus 79% were assessed for ART eligibility, 85% versus 92% initiated ART and 68% versus 66% were retained 12 months after ART initiation. Pre-ART attrition decreased from 61% pre-clinic to 50% post-clinic (*p*<0.001). Pre-ART attrition was associated with being female (sub-distributional hazard ratio (sHR): 1.59; CI: 1.31–1.93), syphilis diagnosis (sHR: 1.47; CI: 1.16–1.85) and slum residence (sHR: 0.84; CI: 0.72–0.97). ART attrition was associated with syphilis diagnosis (hazard ratio (HR): 2.23; CI: 1.35–3.68) and CD4 <50 cells/µL (HR: 1.88; CI: 1.15–3.06).

**Conclusions:**

Implementation of a youth-friendly adolescent clinic improved retention in HIV care among adolescents, particularly in the assessment of ART eligibility and ART initiation. Additional interventions are needed to improve retention among pre-ART patients and support long-term retention among ART patients.

## Introduction

AIDS is the leading cause of death in adolescents and youth in sub-Saharan Africa and the Caribbean [1]. Globally between 2005 and 2012, AIDS-related deaths increased by 50% among adolescents compared to all other age groups, which experienced a 32% decrease [2,3]. Approximately 2.1 million adolescents aged 10 to 19 are living with HIV globally. Each year in resource-poor settings, 900,000 new HIV infections occur among adolescents, representing 40% of all new infections [4].

Haiti is the poorest country and has the highest adult HIV prevalence (1.9%) in the Western Hemisphere [5]. An estimated 140,000 adults and 10,000 adolescents are currently living with HIV in Haiti. Over 40% of all new HIV infections each year in Haiti occur among adolescents, and 80% of new infections are in adolescent girls [6]. Considering the high national adolescent fertility rate (69 per 1000 adolescents and up to 300 per 1000 in the poorest populations), HIV-positive adolescents are at high risk for transmitting HIV to their partners and infants [7]. HIV-positive adolescents must be tested, linked to care and retained in HIV care in order to optimize their own health and reduce further transmission [8,9].

Multiple studies report poor adolescent retention across the HIV care cascade in resource-limited settings [10–17]. One multi-country study in sub-Saharan Africa reported 22% retention among pre-antiretroviral therapy (pre-ART) adolescents and 61% retention among adolescents on ART. Compared to adults, adolescent rates of pre-ART and ART attrition are 50% higher [11]. Recognizing that adolescents, particularly girls, are a high-risk population, the WHO released guidelines in 2013 that emphasized the urgent need to implement adolescent-friendly services to improve retention and outcomes [18].

This analysis describes a decade of adolescent outcomes, before and after the implementation of a youth-friendly adolescent clinic, across the HIV care cascade, from the GHESKIO (Haitian Group for the Study of Kaposi's Sarcoma and Opportunistic Infections) clinic in Port-au-Prince, Haiti. We include pre-ART and ART outcomes using two cascade frameworks and identify risk factors associated with attrition in both periods.

## Methods

### Study design and study population

We conducted a retrospective analysis using programmatic, de-identified, patient-level data from adolescents (aged 13–19), who received an HIV test at GHESKIO between January 1, 2003, and December 31, 2012. Follow-up data were included through December 31, 2014.

### Adolescent HIV services at GHESKIO

GHESKIO is a public HIV clinic in Haiti and the largest provider of HIV treatment services in the Caribbean [19]. In 2003, GHESKIO began providing ART free-of-charge to eligible patients. All HIV services follow WHO and national guidelines [20,21]. Per national guidelines, parental consent and adolescent assent were provided for HIV testing. Adolescents who presented for HIV testing also receive comprehensive screening for other infections including syphilis and tuberculosis (TB). Those who test positive for HIV are referred for HIV care within seven days. Upon enrolment in care, ART eligibility is determined by physical examination and CD4 count testing. ART eligibility criteria from 2003 to 2008 included an AIDS-defining illness or CD4 count <200 cells/µL and changed to a CD4 count <350 cells/µL in 2009 [22,23]. ART-eligible patients receive counselling and initiate ART by a physician. From 2003 to 2010, first-line regimens were zidovudine, lamivudine and efavirenz or nevirpine, with single-drug substitutions permitted as outlined by the WHO [24]. Tenofovir-based regimens became the first-line treatment in 2010. Second-line regimens (lopinavir/ritonavir and atazanavir/ritonavir) have been available since 2004 [23]. Adolescents on ART are followed every month for the first three months and then quarterly by a physician. Adolescents who are not eligible for ART or have not started ART (i.e. pre-ART patients) are followed every three months. Each pre-ART visit involves a medical evaluation for ART eligibility. All patients have CD4 counts performed every six months; viral load is not routinely measured. Retention and adherence activities include peer counselling, transport support, social support programmes and reminder phone calls. Field workers routinely conduct case-finding among patients who are lost from the programme. Dates of ascertained deaths and transfers are entered into the medical record. Data on mode of HIV transmission were not routinely collected.

### Adolescent HIV clinic

From 2003 to 2009, adolescents aged 10–13 were treated in the paediatric clinic and those aged 14–19 in the adult clinic. Services for adolescents in the adult clinic included counselling by peer educators who were HIV-positive patients aged >21, reminder phone calls for appointments and missed appointments, and transport support. Field workers tracked patients who missed appointments. Family planning and treatment for sexually transmitted infections were provided in separate clinics within the GHESKIO campus. In 2009, a dedicated adolescent HIV clinic for patients aged 13–19 was opened in a separate physical space. Staff were trained to provide adolescent-friendly services. Retention and adherence activities were strengthened to specifically address adolescents’ needs. For example, salaried HIV-positive peer counsellors (aged 16–21) provided one-on-one and small group counselling for HIV-positive adolescent patients in the clinic, and age-appropriate educational materials and extracurricular activities such as art projects and games were developed to promote retention. Family planning and sexually transmitted infection testing and treatment were provided within the adolescent clinic. Further, counselling services for mental health and gender-based violence were available at the adolescent clinic. Transport reimbursement and reminder phone calls for appointments and field workers patient tracking continued. Finally, adolescent clinic hours spanned weekdays from 8 am to 5 pm to accommodate after school hours.

### Clinical measurements and outcomes

Demographic measurements included age, sex, education level, number of children and residence zone. Clinical measurements included weight (underweight defined as <25th percentile for weight by gender), CD4 count, WHO stage and co-infection with TB or syphilis. Dates for HIV testing, result of CD4 counts, ART initiation and all clinic and pharmacy visits were documented in the electronic medical record. Data were entered by clinic staff into a central electronic database, de-identified and converted into a Microsoft Excel spreadsheet.

Lost-to-follow-up (LTF) was defined as no clinic or pharmacy visit for six months with no documented transfer or death. Patients who met the definition of LTF were assigned a date of LTF as their last visit. Death and transfers were ascertained from patient records. Patients who transferred were censored on the date of transfer. Re-entry in care was defined as a patient who was categorized as LTF but returned to care at a later time.

The binary outcomes for multivariable regression were attrition before ART initiation at 12 months (±3 months) after HIV testing for pre-ART patients and attrition at 12 months (±3 months) after ART initiation for ART patients. Attrition was defined as LTF or dead.

Two HIV care cascade frameworks are used to evaluate pre-ART and ART outcomes before and after implementing the adolescent clinic (2003–2009 pre-clinic and 2009–2013 post-clinic). The first is the traditional HIV treatment cascade that includes (1) testing HIV positive defined as a positive serologic HIV test, (2) enrolment in HIV care defined as at least one clinical visit, (3) assessment of ART eligibility defined as a documented CD4 count and/or WHO stage, (4) ART initiation defined as date of first ART prescription pick-up and (5) ART retention at 12 months from date of ART initiation or documented transfer to another facility. Of note, this approach does not capture outcomes of pre-ART patients who are retained in care. The second is the comprehensive HIV care cascade, which has been described in prior reports and evaluates pre-ART in addition to ART outcomes [24]. This alternative cascade uses a longitudinal cohort approach to classify outcomes for all patients over 12 months from time of HIV testing. Outcomes are classified into three mutually exclusive categories, which were optimal, suboptimal and poor and were measured at three, six and 12 months from HIV testing. Optimal outcomes include retained in pre-ART care with known ART ineligibility, initiated ART, retained on ART or documented transfer. Suboptimal outcomes include retained in pre-ART care with known ART eligibility and retained in pre-ART care with undocumented CD4 count or indeterminate WHO stage. Poor outcomes include LTF and death.

### Statistical analysis

We calculated the number and proportion of patients retained at each step in the HIV treatment cascade and the number and proportion of patients achieving each category (optimal, suboptimal and poor) in the comprehensive HIV care cascade at three, six and 12 months from HIV testing. We calculated sub-distributional hazard ratios (sHRs) for attrition in the pre-ART period treating pre-ART attrition and ART initiation as competing risks as outlined by Fine and Grey [25]. Among patients who initiated ART, hazard ratios (HRs) for rates of attrition at 12 months from ART initiation were calculated using Cox proportional hazards models. Statistical analyses were conducted using Stata 14 (Stata Corp. College Station, Texas, USA) and SAS 9.3 (SAS Institute, Cary, NC, USA).

### Ethics

This analysis was approved by the Institutional Review Boards at GHESKIO, Weill Cornell Medical College and Columbia University.

## Results

### Patient characteristics

Between January 1, 2003 and December 31, 2012, a total of 41,218 adolescents were tested for HIV, and 1672 (4.1%) adolescents tested HIV positive. Over the 10-year study period, the number of adolescents testing HIV positive ranged from 120 in 2003 to 207 in 2012. HIV prevalence among those tested declined from 4.6% in 2003 to 3.7% in 2012. Among adolescents who tested HIV positive, 1336 (80%) were female and median age was 18 years (IQR 16–19) (Table 1). The majority reported no schooling or only primary schooling and nearly half lived in the slum communities surrounding GHESKIO. Twenty-two percent had children. At the time of testing, 354 (21%) adolescents reported current use of a modern family planning method. Ten percent of adolescents had syphilis at the time of HIV testing. Median CD4 count at enrolment in care was 414 cells/µL (IQR 238–604), with 53% not having a CD4 within 90 days of HIV testing due to LTF after HIV testing or lack of CD4 count testing at the clinic. Median CD4 count pre-clinic was 424 cells/µL (IQR 260–602) versus 401 cells/µL (IQR 197–605) post-clinic (*p*=0.11).

**Table 1:** HIV-infected adolescent patient characteristics at HIV testing, aged 13 to 19, in Haiti from 2003 to 2012

	At HIV testing (*N=*1672)	
	
	*N*	%
Sex		
Male	336	20
Female	1336	80
Age at HIV testing		
Median age, years	18	IQR (16–19)
13 to 15	287	17
16 to 19	1385	83
Education		
None/some primary	956	57
≥Some secondary	716	43
Residence zone		
Within Port-au-Prince with slum	794	47
Within Port-au-Prince without slum	648	39
Outside of Port-au-Prince	94	6
Not recorded	136	8
Number of children		
0	1304	78
1+	368	22
Use of family planning		
Yes	354	21
No	1318	79
CD4 count (cells/µL) at the time of enrolment		
Median	414	IQR (238–604)
<50	72	4
51 to 200	93	6
201 to 350	146	9
>351	474	28
Not recorded	887	53
Active tuberculosis^a^		
Yes	19	1
No	1653	99
Syphilis diagnosis^b^		
Positive	172	10
Negative	1500	90
Year of testing		
2003	120	7
2004	151	9
2005	174	11
2006	162	10
2007	180	11
2008	175	10
2009	184	11
2010	150	9
2011	169	10
2012	207	12

a Tuberculosis diagnosis within six months of HIV testing

b syphilis diagnosis within six months of HIV testing.

Table 2 describes the characteristics of the 554 adolescents who initiated ART during the study period, which increased from 10 patients in 2003 to 146 in 2012. Median CD4 count at the time of ART initiation was 251 cells/µL (IQR 104–373). Sixty-two percent of patients had WHO stage III/IV symptoms. Median weight at ART initiation was 51 kg (IQR 40–57) among males and 48 kg (IQR 40–56) among females.

**Table 2:** HIV-positive adolescent patient characteristics at antiretroviral therapy initiation

	At ART initiation^a^ (*N*=554)	
	
	*N*	%
Sex		
Male	100	18
Female	454	82
Age at ART initiation		
Median age	19	IQR (17–20)
13 to 15	90	16
16 to 19	309	56
20+	155	28
Education		
None/some primary	325	59
≥Some secondary	229	41
Residence zone		
Within Port-au-Prince with slum	268	48
Within Port-au-Prince without slum	212	38
Outside of Port-au-Prince	32	6
Not recorded	42	8
Number of children		
0	431	78
1+	123	22
Weight at ART initiation		
Median male	51	IQR (40–57)
Median female	48	IQR (40–56)
Underweight (<lowest quartile by gender)	128	23
Normal weight (≥lowest quartile by gender)	393	71
Not recorded	26	5
CD4 count (cells/µL)at the time of ART initiation		
Median	251	IQR (104–373)
<50	66	12
51 to 200	120	22
201 to 350	179	32
>351	155	28
Not recorded	34	6
WHO stage at ART initiation		
1/2	183	33
3/4	345	62
Not recorded	26	5
Active tuberculosis^b^		
Yes	15	3
No	539	97
Syphilis diagnosis^c^		
Positive	35	6
Negative	519	94
ART regimen at initiation		
First line: zidovudine, lamivudine, efavirenz or nevirapine	307	55
First line: tenofovir-based regimen^d^	215	39
First line: other regimen^e^	30	6
Second line: lopinavir-ritonavir regimen	2	<1
Year of ART initiation		
2003	10	2
2004	13	2
2005	17	3
2006	9	2
2007	49	9
2008	36	7
2009	62	11
2010	55	10
2011	102	18
2012	146	26
2013^f^	55	10

a Characteristics of patients in Table 1 at ART initiation

b tuberculosis diagnosis within six months of ART initiation

c syphilis diagnosis within six months of ART initiation

d stavudine, didanosine, abacavir (ABC)

e zidovidine, emtricitabine, neverapine, efaverinz

f January to June 2013. ART, antiretroviral therapy.

### HIV care cascades

**Figure 1:**
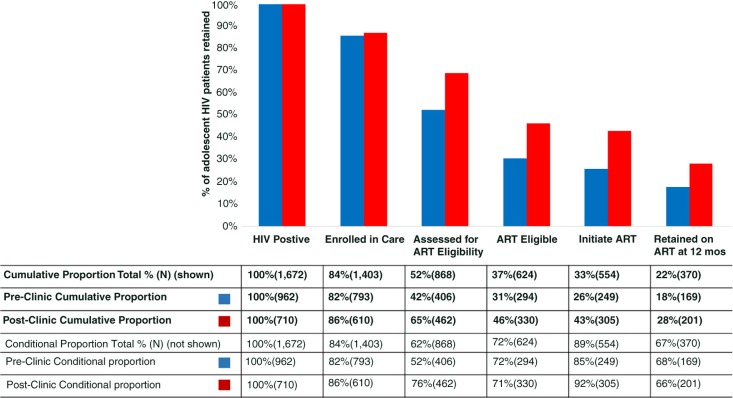
HIV treatment cascade for 1672 adolescents aged 13 to 19 in Haiti from 2003 to 2012.

Figure 1 compares outcomes before and after implementation of the adolescent clinic assessing each step of the traditional HIV treatment cascade. By step: 82% (793/962) versus 86% (610/710) of HIV-positive patients enrolled in care (*p*=0.41); 42% (406/793) versus 76% (462/610) of those enrolled were assessed for ART eligibility with CD4 count testing (*p*<0.0001); 85% (249/294) versus 92% (305/330) of eligible patients initiated ART (*p*<0.0001); and 68% (169/249) versus 66% (201/305) of patients who initiated ART were retained in care at 12 months (*p*=0.5). Cumulatively, a total of 28% (201/710) of all adolescents who tested HIV positive were retained in care 12 months after ART initiation compared to 18% (169/962) pre-clinic (*p*<0.001).

**Figure 2:**
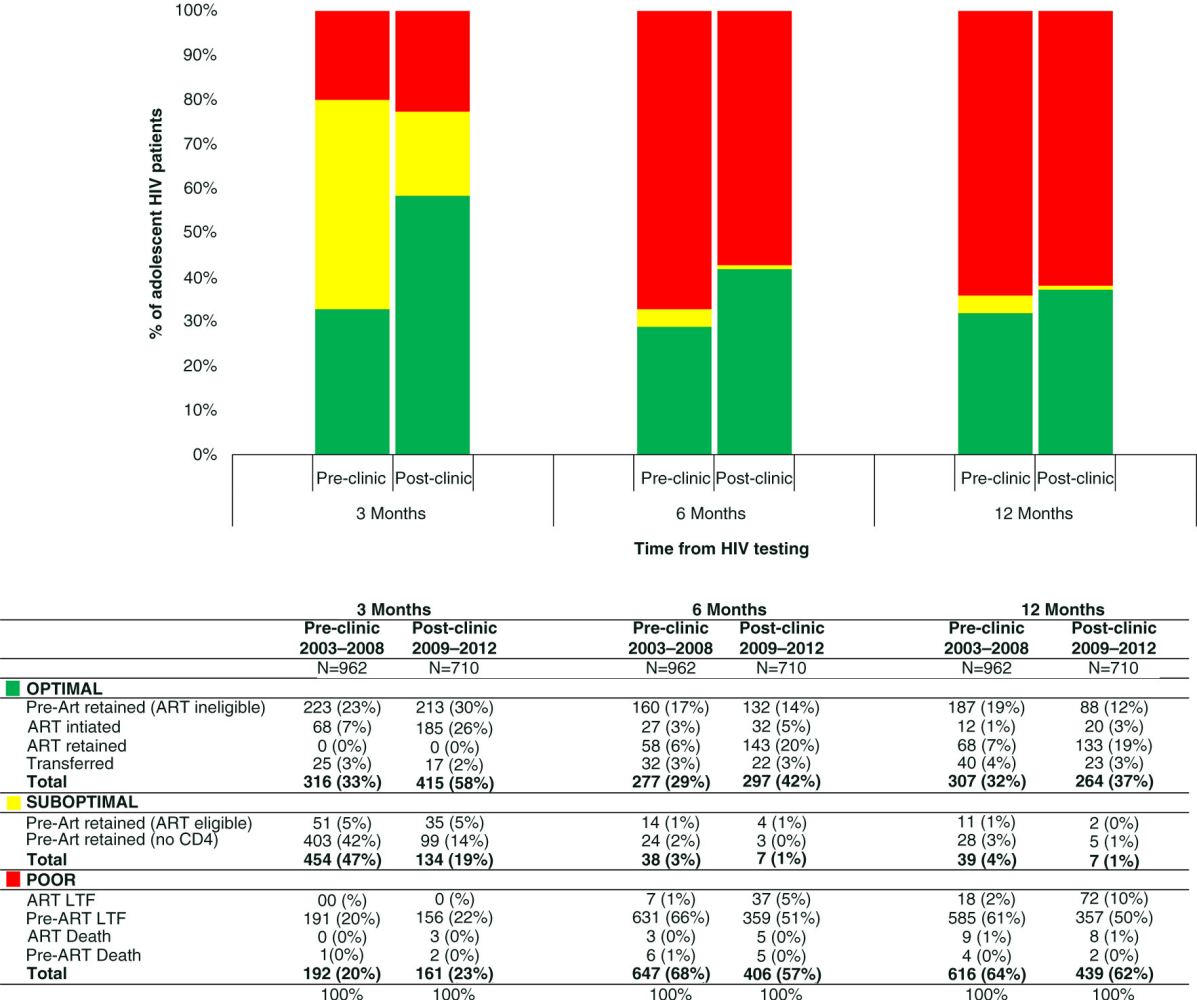
Comprehensive HIV care cascade for 1672 adolescents aged 13 to 19 in Haiti from 2003 to 2012: outcomes at three, six and 12 months after HIV testing.

The comprehensive HIV care cascade (Figure 2) includes outcomes on 1118 pre-ART patients (67% of all patients who tested HIV positive) not captured in the HIV treatment cascade. The proportion of patients with optimal outcomes was higher after the implementation of the adolescent clinic at all time periods. At three months, patients with optimal outcomes increased from 33% (316/962) pre-clinic to 58% (415/710) post-clinic; at six months, from 29% (277/962) pre-clinic to 42% (297/710) post-clinic; and at 12 months, from 32% (307/962) pre-clinic to 37% (264/710) post-clinic. The largest increase in a subcategory of optimal outcomes at 12 months was among patients retained on ART, 7% (68/962) of patients pre-clinic compared to 19% (133/710) post-clinic.

The proportion of patients categorized with suboptimal outcomes decreased post-clinic. At three months, 47% ( 454/962) of adolescents had suboptimal outcomes pre-clinic compared to 19% (134/710) post-clinic. The largest subcategory of suboptimal patients at three months were pre-ART patients without a CD4 count, which decreased from 42% (403/962) pre-clinic to 14% (99/710) post-clinic. The proportion of patients who were ART eligible but had yet to initiate treatment remained 5% of all patients pre- and post-clinic (51/962 and 35/710, respectively). The median CD4 count of these ART-eligible patients was 59 cells/µL (IQR 23–142) pre-clinic and 249 cells/µL (IQR 144–298) post-clinic. Among the 454 patients with suboptimal outcomes at three months pre-clinic, 398 (88% of suboptimal patients) were LTF or dead at 12 months. Among the 134 patients with suboptimal outcomes at three months post-clinic, 113 (84% of suboptimal patients) were LTF or dead at 12 months.

The proportion of patients with poor outcomes at three months was similar pre- and post-clinic, 20% (192/962) and 23% (161/710), respectively. At six months, the proportion of patients with poor outcomes increased to 67% (647/962) pre-clinic and 57% (406/710) post-clinic, with little change at 12 months, 64% (616/962) and 62% (439/710), respectively. The largest subcategory of patients with poor outcomes were pre-ART patients who were LTF. This subcategory decreased from 66% (631/962) pre-clinic to 51% (359/710) post-clinic at six months and 61% (585/962) pre-clinic to 50% (357/710) post-clinic at 12 months. LTF among ART patients increased from 2% (18/962) pre-clinic to 10% (72/710) post-clinic. Pre-clinic, there were 13 deaths (1.3% of all patients) – nine ART and four pre-ART patients. Post-clinic, there were 10 deaths (1.4% of all patients) – eight ART and two pre-ART patients.

### Re-entry in care

The total number of patients categorized as LTF at 12 months in the comprehensive HIV cascade (Figure 2) was 1032 (18 ART patients and 585 pre-ART patients pre-clinic plus 72 ART patients and 357 pre-ART patients post-clinic). Among those LTF at 12 months, a total of 19% (198/1032) of patients re-entered care with at least one clinic visit in the study period after having been defined as lost (Table 3). Median time to re-entry was 25 months from last clinic visit. Approximately half re-entered care between 12 and 24 months – 46% (91/198), and half after 24 months – 54% (107/198). A total of 51% (100/198) of patients who re-entered care subsequently started ART with a median time to initiating ART from HIV testing of 42 months. The vital status among patients who re-entered care at the time of censor for this study included 20% (40/198) of patients alive in care, 76% (150/198) of patients LTF and 4% (8/198) of patients died.

**Table 3:** Re-entry in care among patients lost-to-follow-up

	Among patients categorized as LTF^a^	
	
	12 months after HIV testing (*N*=1032)	12 months after ART initiation (*N*=184)
	
	*N* (%)	*N* (%)
Re-entered care	198 (19)	35 (19)
Re-entered and initiated ART	100 (10)	N/A
	**Among patients who re-entered care^b^**	
	**12 months after HIV testing** **(*****N*****=198)**	**12 months after ART initiation** **(*****N*****=35)**
	***N*****(%)**	***N*****(%)**
Alive and in care	40 (20)	6 (17)
LTF	150 (76)	26 (74)
Transferred	0 (0)	1 (3)
Died	8 (4)	2 (6)

a LTF defined as no clinic or pharmacy visit for six months with no documented transfer or death

b re-entry defined as a patient categorized as LTF but who returned for at least one clinic visit at a later time. LTF, lost-to-follow-up; ART, antiretroviral therapy.

The total number of patients on ART who were LTF in the HIV treatment cascade (Figure 1) was 184 (554 initiated ART minus 370, who were retained at 12 months from initiation). Among the 184 patients who were LTF at 12 months from ART initiation, 19% (35/184) returned for at least one clinic visit in the study period. Median time to re-entry was 21 months from last clinic visit; 69% (24/35) of patients re-entered care between 12 and 24 months from date of ART initiation and 31% (11/35) of patients re-entered care after 24 months. The vital status at the time of censor for this study of the 35 ART patients who re-entered care included 17% (6/35) of patients alive and in care, 74% (26/36) of patients LTF, 6% (2/35) of patients died and 3% (1/35) transferred.

### Factors associated with attrition in pre-ART and ART care

Table 4 presents variables associated with attrition in the first year of pre-ART and ART care. In adjusted analyses of pre-ART attrition, females had higher attrition compared to males (sHR: 1.59; CI: 1.31–1.93), patients with a positive syphilis diagnosis at the time of enrolment had higher attrition (sHR: 1.47; CI: 1.16–1.85) and patients testing HIV positive before the adolescent clinic had higher attrition (sHR: 1.44; CI: 1.24–1.69). Patients living in a slum area compared to non-slum areas had lower attrition (sHR: 0.84; CI: 0.72–0.97). In adjusted analyses of attrition from ART care, patients with a CD4 count <50 cells/µL had higher attrition than patients with CD4 count >350 cells/µL (HR: 1.88; CI: 1.15–3.06), patients with a positive syphilis diagnosis at ART initiation had higher attrition (HR: 2.23; CI: 1.35–3.68) and patients with missing weight at ART initiation had higher attrition than those with normal weight (>25th percentile of weight by gender) (HR: 2.61; CI: 1.44–4.76).

**Table 4:** Multivariable regression: factors associated with attrition from pre-ART care and ART^a^

	Pre-ART^b^				ART^c^			
		
	Unadjusted		Adjusted^d^		Unadjusted		Adjusted^d^	
	sHR (95% CI)	*p*	sHR (95% CI)	*p*	HR (95% CI)	*p*	HR (95% CI)	*p*
Sex								
Female	**1.54 (1.27, 1.87)**	**<0.001**	**1.59 (1.31, 1.93)**	**<0.001**	0.98 (0.71, 1.36)	0.978	1.08 (0.76, 1.54)	0.662
Male	1		1		1		1	
Age								
16 to 19	0.98 (0.81, 1.190)	0.866	0.93 (0.76, 1.15)	0.511	1.17 (0.78, 1.76)	0.448	1.07 (0.69, 1.66)	0.770
13 to 15	1		1		1		1	
Education								
Primary	1.00 (0.87, 1.16)	0.992	1.01 (0.87, 1.17)	0.946	0.94 (0.70, 1.26)	0.673	0.95 (0.70, 1.29)	0.728
Secondary	1		1		1		1	
Year								
Pre-clinic	**1.40 (1.20, 1.63)**	**<0.001**	**1.44 (1.24, 1.69)**	**<0.001**	0.89 (0.66, 1.19)	0.424	0.83 (0.60, 1.13)	0.234
Post-clinic	1		1		1		1	
Number of children								
1+	1.00 (0.84, 1.19)	0.984	0.93 (0.85, 1.21)	0.919	1.24 (0.89, 1.73)	0.206	1.27 (0.89, 1.80)	0.186
0	1		1		1		1	
Residence								
PAP w/out slum	1		1		1		1	
PAP with slum	**0.84 (0.72, 0.98)**	**0.028**	**0.84 (0.72, 0.97)**	**0.021**	1.11 (0.80, 1.52)	0.542	1.09 (0.79, 1.51)	0.587
Outside of PAP	0.84 (0.59, 1.19)	0.325	0.85 (0.60, 1.20)	0.345	1.57 (0.88, 2.79)	0.128	1.72 (0.95, 3.13)	0.074
Not recorded	1.04 (0.79, 1.40)	0.764	1.04 (0.76, 1.39)	0.799	1.23 (0.70, 2.16)	0.471	1.08 (0.61, 1.92)	0.796
Syphilis diagnosis								
Positive	**1.34 (1.06, 1.69)**	**0.014**	**1.47 (1.16, 1.85)**	**0.001**	**2.27 (1.43, 3.62)**	**<0.001**	**2.23 (1.35, 3.68)**	**0.002**
Negative	1		1		1		1	
Active TB								
Positive	**0.27 (0.09, 0.82)**	**0.021**	0.32(0.10, 1.01)	0.054	0.98 (0.40, 2.39)	0.969	0.67 (0.26, 1.70)	0.403
Negative	1		1		1		1	
CD4 count								
Missing	–	–	–	–	1.58 (0.90, 2.76)	0.112	1.32 (0.74, 2.39)	0.345
<50	–	–	–	–	**1.75 (1.13, 2.70)**	**0.012**	**1.88 (1.15, 3.06)**	**0.011**
51 to 200	–	–	–	–	0.82 (0.53, 1.27)	0.379	0.95 (0.59, 1.31)	0.812
201 to 350	–	–	–	–	0.81 (0.55, 1.19)	0.283	0.88 (0.59, 1.31)	0.525
>350	–	–	–	–	1		1	
Weight (kg)								
Normal weight	–	–	–	–	1		1	
Underweight^e^	–	–	–	–	0.98 (0.68, 1.40)	0.894	0.89 (0.61, 1.29)	0.541
Not recorded	–	–	–	–	**3.12 (1.88, 5.19)**	**<0.001**	**2.61 (1.44, 4.76)**	**0.002**
WHO stage								
3/4	–	–	–	–	1		1	
1/2	–	–	–	–	0.88 (0.64, 1.21)	0.876	0.83 (0.58, 1.19)	0.310
Not recorded	–	–	–	–	**1.98 (1.14, 3.46)**	**0.016**	1.21 (0.63, 2.32)	0.562

a Pre-ART attrition assessed at 12 months from HIV testing, and ART attrition assessed at 12 months from ART initiation

b characteristics at HIV testing. Weight, WHO stage and CD4 not regularly recorded at HIV testing

c characteristics at ART initiation

d controlled for sex, age (13–15 and 16–19), education level (completion of some or all primary school, completion of some or all secondary school), year of enrolment/ART initiation, number of children, CD4 count at enrolment/ART initiation, weight at ART initiation, prevalent diagnosis at enrolment/ART initiation, active TB at enrolment/ART initiation, WHO stage at ART initiation and residence code (Port-au-Prince area with a slum, Port-au-Prince area without a slum, outside of Port-au-Prince)

e underweight defined as <25th percentile for weight. ART, antiretroviral therapy.

The 2010 earthquake did not affect outcomes for ART or pre-ART patients, and risk factors for attrition did not change in the post-earthquake period (data not shown).

## Discussion

The implementation of a youth-friendly adolescent HIV clinic improved outcomes among both pre-ART and ART patients by increasing the proportion of patients enrolling in HIV care, being assessed for ART eligibility and initiating ART. Additionally, pre-ART attrition decreased from 61% pre-clinic to 50% post-clinic. Our results emphasize that implementation of youth-friendly HIV services can improve outcomes, particularly in the first six months after HIV testing; however, pre-ART attrition remains extremely high and more work is needed to engage asymptomatic patients and promptly initiate ART-eligible patients on treatment. The finding that youth-friendly clinic services did not impact long-term retention of ART patients underscores the multiplicity of socio-economic, family and community factors, which acutely influence adolescent behaviour in addition to clinic-level factors [26,27].

Interestingly, the adolescent clinic improved retention in the early cascade steps when clinical services are the focus, for example, HIV testing, blood draws for a CD4 count and ART initiation. These improvements could be explained by streamlined care in the adolescent clinic as adolescents received testing, treatment and laboratory procedures in one location. The dedicated space also may have reduced stigma and increased confidentiality given that patients no longer mix with adults. Finally, training staff in youth-friendly care may have increased acceptability of services. However, long-term retention (both pre-ART and ART), which is highly influenced by social factors such as stigma, isolation and lack of psychosocial support, and economic factors such as access to transport fees and food remain challenges that are not easily influenced by clinic-based interventions [28–30]. These social, behavioural and structural barriers are exacerbated by the emotional, psychological and physical changes that occur during adolescence, creating a “perfect storm,” which impedes long-term retention.

Our results were similar to a study in Kenya, which reported that youth-friendly HIV services did not improve long-term retention [31]. In comparison to a study on retention among adults in HIV care at GHESKIO [32], a higher proportion of adolescents in our cohort were LTF at each step in the cascade. In the adult cohort, 91% returned for an HIV test result compared to 86% of adolescents; 84% of adults returned for a CD4 count compared to 69% of adolescents; and 94% of eligible adults initiated ART compared to 89% of adolescents.

Additional interventions beyond clinic-based services are needed to address long-term retention. Interventions may target social and behavioural barriers, facilitate family disclosure and encourage family support [33]. Studies suggest strengthening adolescents’ social networks improves retention by reducing isolation and HIV-related stigma, and improving family relations [34–36]. Providing services in community-based programmes outside of the clinic could reduce the stigma of attending an HIV clinic and alleviate transportation costs. In our study, living in a slum area was associated with significantly lower attrition compared to non-slum areas. GHESKIO community outreach activities directly target slum communities, which could explain the lower proportion of loss and provide evidence for increasing community-based services. Individual case-management could provide essential social support and uniquely tailor services to strengthen follow-up during chaotic peaks in an adolescent's life (e.g. when a parent or guardian dies, or the adolescent is displaced from the home).

Although pre-ART attrition decreased post-clinic and is comparable to other adolescent HIV cohorts in sub-Saharan Africa, it remains far from optimal. One study in Kenya reported 44% pre-ART attrition among adolescents, which compares to 51% in our study [13]. Among patients with poor outcomes at 12 months, 95% pre-clinic and 83% post-clinic were pre-ART patients. The majority of pre-ART attrition occurs within the first six months of care, prior to assessment of ART eligibility. The results of the HIV comprehensive care cascade show that a majority of patients who were retained in pre-ART care for the first three months but did not have a CD4 count (nearly 50% of patients pre-clinic and nearly 20% of patients post-clinic) were ultimately lost by 12 months. Same-day CD4 testing with point-of-care equipment could greatly improve the proportion of patients who are assessed for ART eligibility, found to be eligible and initiated on ART, thus shifting pre-ART patients to ART care. Since 2015, the adolescent clinic has adopted point-of-care CD4 testing in an effort to improve this step. Additionally, low CD4 counts of ART-eligible patients who were retained but did not initiate treatment underscore the importance of prompt ART initiation. GHESKIO has begun fast-track ART initiation to remediate this. The fact that nearly 20% of pre-ART patients who were LTF at 12 months re-enter care and 50% of those who re-enter initiate ART suggests that asymptomatic teens are lost only to return with more advanced disease, indicating the importance of preventing interruptions in adolescent HIV care. Our adolescent re-entry rates appear similar to adult re-entry rates reported from developing countries [37]. Further analyses are needed to determine the impact of interruptions in care on health outcomes and HIV transmission among adolescents. A shift to universal ART could decrease pre-ART attrition and interruption in care by initiating all patients.

This study has several strengths. We present 10 years of outcomes beginning from the first time ART was offered in Haiti and report the impact of a WHO-recommended intervention aimed at improving adolescent HIV care. We also present data on linkage from HIV testing to enrolment in HIV care. Few studies are able to capture linkage from HIV testing to enrolment in care because longitudinal data are often missing at this step [11,13,38]. Our results show a high percentage of adolescents link to care after HIV testing, 86% in our study linked compared to 62 and 63% in two studies in similar settings [39,40]. Pre-ART attrition data are limited, particularly among adolescents. This study shows that the highest adolescent attrition occurs during the pre-ART period. Finally, while the majority of participants of HIV-positive adolescent cohort studies are also female [11,13,28,41–43], our cohorts’ age is older (aged 16–19) and unique in that HIV transmission is likely via horizontal transmission and likely heterosexual transmission, not perinatal transmission. Studies in resource-poor settings often follow vertically infected children transitioning to adolescent and adult care [17,44]. It is important to evaluate heterosexually infected adolescents as risk factors and interventions may differ.

Limitations of this study include that it was conducted at one clinic in Haiti, albeit the largest HIV treatment clinic in the country, and results may not be generalizable to all HIV-infected adolescent populations. Second, optimal HIV care includes viral load suppression, but viral load measurement was not widely available in Haiti until 2015, thus we are unable to report on the adolescent clinic impact on viral load suppression.

## Conclusions

This 10-year cohort study provides data to support the implementation of youth-friendly HIV services, a WHO-recommended intervention, to improve retention in care among HIV-positive adolescents. Implementation of a dedicated adolescent clinic improved retention immediately after HIV testing, assessment for ART eligibility and ART initiation, but did not increase retention after ART initiation. Additional interventions beyond youth-friendly clinic services are needed to improve long-term retention, optimize outcomes among adolescents living with HIV and curb a growing global epidemic among adolescents.
